# Supplementary material for: Indoor air surveillance and factors associated with respiratory pathogen detection in community settings in Belgium
Source: Nat Commun. 2023 Mar 11;14:1332. doi: 10.1038/s41467-023-36986-z (PMC10005919; doi:10.1038/s41467-023-36986-z)
Supplement: Supplementary file 1 — Supplementary Information [file 41467_2023_36986_MOESM1_ESM.pdf]

Supplementary Information

Supplementary Methods

Validation of the respiratory panel in clinical practice

The in-house developed 29-pathogen respiratory panel has been in use by the clinical laboratory of University Hospitals Leuven since 2016. The primer and probe sequences used in the current panel, consisting of 12 multiplex PCRs, are listed in *Supplementary Table 12.* This laboratory is the largest clinical diagnostic laboratory in Belgium and is the National Reference Laboratory for respiratory viruses. The respiratory panel is accredited by the Belgian Federal Accreditation Body BELAC[1] under ISO15189:2012 and is thus subjected to continuous validation using External Quality Control (EQC) samples, cultures and clinical samples, as per *Rabenau et al*[2]. The *Methods* section of the main article outlines the Internal Qualiry Control (IQC) samples which are analysed with the respiratory panel. In addition, *Supplementary Table 12* lists the highest level of validation to which each pathogen specific qPCR was subjected in the past 5 years, and the clinical cutoffs, which were derived from those validation experiments. We highlight several additional experiments underlining the validity of the panel.

One experiment, published as a conference abstract, validated the in-house respiratory panel against 3 Panther Fusion respiratory panel assays (Panther Fusion Flu A/B/RSV, Panther Fusion Paraflu and Panther Fusion AdV/hMPV/RV, HOLOGIC®) on 245 clinical respiratory samples for 10 respiratory viruses: influenza A virus, influenza B virus, respiratory syncytial virus A/B, *Human metapneumovirus,* human adenovirus, human enterovirus (incl. rhinovirus), human parainfluenza viruses 1 to 4. The agreement varied between 95 and 99% for each of these pathogens[4].

Another unpublished experiment validated the in-house respiratory panel against 18 matching pathogens in the QIAstat-Dx Respiratory SARS-CoV-2 Panel (QIAGEN®): influenza A virus, influenza B virus, respiratory syncytial virus A/B, *Human metapneumovirus*, human parainfluenza viruses 1 to 4, human adenovirus, human enterovirus (incl. rhinovirus), human bocavirus, *Human coronaviruses 229E, HKU-1, NL63* and *OC43,* SARS-CoV-2*, Mycoplasma pneumoniae*, *Legionella pneumophila)*. Out of 36 samples, 4 were negative, 30 were pooled samples consisting of 1 to 4 samples known to be positive for multiple pathogens (based on prior detection using the in-house respiratory panel) and 2 samples were first-line controls consisting of pooled DNA/RNA extracts of different pathogens. The overall positive percentage agreement for all pathogens between the panel and the known positivity of samples was 97.5% (119/122) for the in-house respiratory panel and 79.5% (97/122) for the QIAstat-Dx Respiratory SARS-CoV-2 Panel (QIAGEN®). The overall agreement between both panels was 95.7% (620/648). The pathogens which were only detected in one of both panels - 3 were only positive in the QIAstat-Dx Respiratory SARS-CoV-2 panel and 22 were only positive in the in-house respiratory panel - generally had high Ct values (internal results communicated by co-author Kurt Beuselinck).

At the start of the SARS-CoV-2 pandemic, the existing primer-probe mix for SARS-CoV-1 was updated as the first 6 published Wuhan strain sequences had 2 mismatches with the existing forward primer, 3 mismatches with the existing reverse primer and 1 mismatch with the existing probe. They were later verified against additional published sequences using BLAST alignment. A new validation of the corresponding multiplex qPCR was performed on dilutions of a SARS-CoV-1 culture. When comparing both panels on 6 SARS-CoV-1 samples, two of which with Ct values above 33.0, and 40 negative samples, there was 100% concordance between both PCRs.

In May 2020, the test was also validated for SARS-CoV-2 detection (*Supplementary Table 11*). The SARS-CoV-1/2 qPCR form the respiratory panel, aimed at ORF1ab, was compared against a laboratory developed E-gen RT-qPCR. Both used 5 µl extract and were run on Quantstudio Dx (Thermo Fisher Scientific, Waltham, MA). Tests were run on QCMD samples also used in the Coronavirus disease (COVID-19) outbreak preparedness EQA pilot study[5], a commercially sourced sample (Qnostics®), a strong positive clinical sample (Wuhan strain) and dilutions from a heat-inactivated SARS-CoV-2 culture from that sample, which was used in a national effort to standardize SARS-CoV-2 diagnosis and reporting by Belgian clinical laboratories[6]. The results are shown in *Supplementary Table 11.* In all but one of these tests (the Qnostics sample 250 c/ml), the qualitative result was the same for both qPCRs. However, Ct values were consistently higher in the respiratory panel SARS-CoV-2 qPCR, which is consistent with the lower sensitivity of this qPCR than the TaqPath qPCR on air samples in the current study.

Investigating the influence of the qPCR panel on SARS-CoV-2 detection in ambient air samples

We included a second, single pathogen, SARS-CoV-2 qPCR (TaqPath) on top of the ORF1ab directed SARS-CoV-1/2 qPCR in the respiratory panel for several reasons. The first was the epidemiological relevance of SARS-CoV-2. Secondly, we knew that the former did not match the latter in terms of Ct values in clinical diagnostics, suggesting a lower sensitivity (*Supplementary Table 11*). Third, we did have experience screening ambient air for SARS-CoV-2 using the TaqPath assay before the current study, which we did not using the respiratory panel[7].

We did notice a reduced sensitivity of the SARS-CoV-2 qPCR contained in the respiratory panel in our study, as opposed to the TaqPath qPCR. We investigated this difference using only samples with results for both by means of a McNemar test. A two-sided p-value of ≤ 0.05 was

considered significant in all analyses. We did not correct for multiple testing. See *Supplementary Table 10*.

Excluding possible non-specific amplification in ambient air samples

When we obtained respiratory panel qPCR Ct values above the clinically defined threshold for a particular pathogen (*Supplementary Table 12*), we repeated the qPCR with fresh primer probe mixes, since an association had been observed between the storage time of frozen aliquoted primer-probe mixes and the number of possible non-specific amplifications in clinical diagnostics. For each pathogen with possible non-specific amplification (air sample Ct values were above the clinically defined threshold), up to five samples with the lowest Ct values were reanalyzed. If at least 60% of samples returned positive, the initial results were retained. If less than 60% of samples returned positive, we reclassified all results of samples with possible non-specific amplification for that pathogen as negative (*Supplementary Table 13*).

Imputing missing environmental/building related and behavioural factors

If environmental/building related factors were lost during data collection, we proceeded as follows to impute their values:

- If a manual measurement was missing at the start or end of sampling, the corresponding other value was used as average for that sample.
- Missing values for temperature were inferred by computing the mean of measurements in the same location in the same period (7 days before to 7 days after). Both manual and continuous measurements were included.

When there was no registered value either before or after sampling for mask wearing, natural ventilation or vocalization, the following methods were used consecutively to infer the value:

- First, the source notes were checked for information to fill the missing datapoint.

- Second, we computed the mean of measurements in the same location in the same period (7 days before until 7 days after) to infer the value.
- Third, the value was inferred from memory by the data entry team.

Supplementary Figures

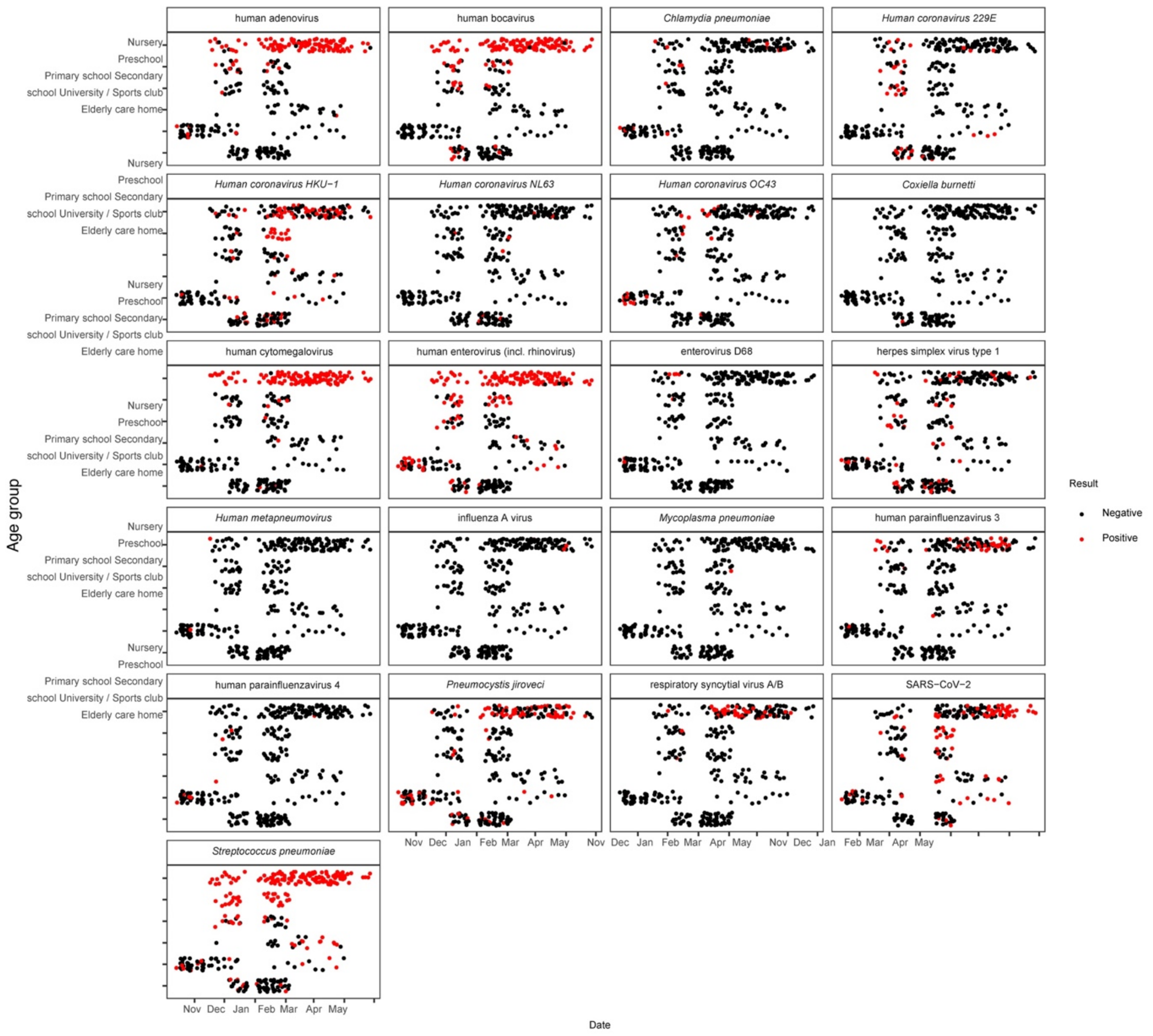

Supplementary Figure 1. Overview of air samples and qPCR results for 29 pathogens. *This figure shows the positive (red) and negative (black) qPCR tests for 29 pathogens on environmental air samples. Pathogens which were positive in at least one air sample are shown. For SARS-CoV-2, it only shows the TaqPath qPCR results. Sample stratification (y axis) is by predominant age group: nursery (0-3y), preschool (3-6y), primary school (6-12y), secondary school (12-18y), adults (18-65y) and nursing homes (65+). The age groups 18y-25y and 25-65y were pooled as the latter represented only a limited number of samples. The x axis*

shows the timing of sampling. In October, sampling only took place in areas populated by university students. This expanded to a nursery, preschool, primary and secondary school in November. In December and January, we added several elderly care homes and one bar of a sports club. Sampling in university locations was reduced from the second part of December onwards due to exams. It paused around the Christmas holidays in schools. Sampling intensified in the nursery environment from January onwards as air filtration was evaluated there.

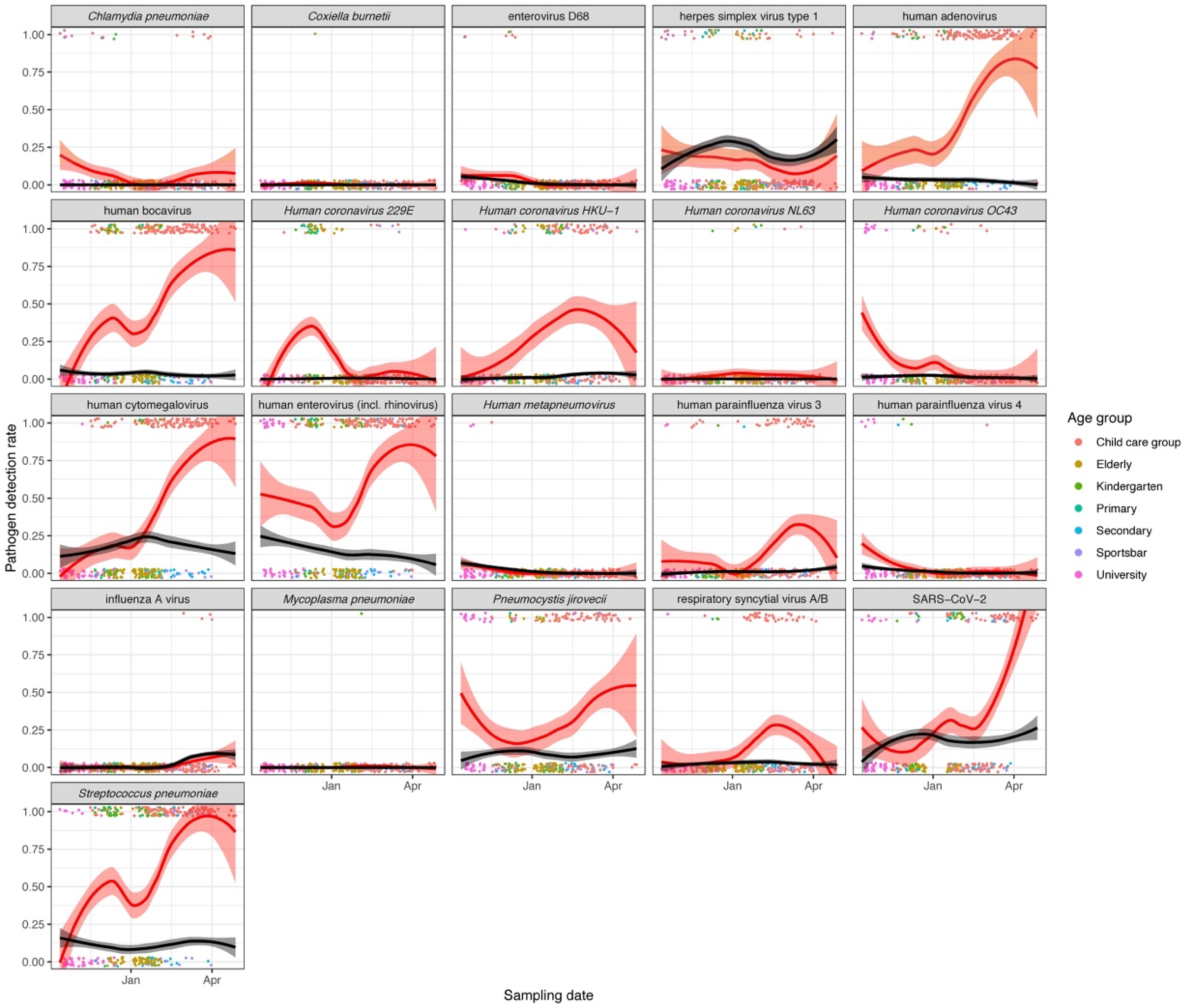

***Supplementary Figure** 2. **Positivity rates of respiratory pathogens in ambient air in all locations.** Each panel represents qPCR test results of one of the 29 targeted pathogens, plotted by sampling date. Each pathogen which was positive in at least one air sample is shown. For SARS-CoV-2, the TaqPath results are shown. Dots represent datapoints of individual ambient air samples (see figure legend for age specific color codes. 0=negative, 1=positive). Red lines and shaded areas show a corresponding LOcally weEighted Scatterplot Smoothing (LOESS) regression model's estimate of the detection rate for each pathogen with 95% confidence*

intervals (t-based approximation), representing the model's estimate in all sampling sites. We included 337 air samples. For comparison, we retrieved the results of the same 29 pathogen multiplex qPCR respiratory panel, performed on 1522 samples from patients of all ages with respiratory infections at University Hospitals Leuven between October 2021 and May 2022. This hospital is adjacent to the nursery and drains most patients in the region. Black lines and shaded areas show a corresponding LOESS regression of the detection rate for each pathogen with 95% confidence intervals (t-based approximation). Individual datapoints are not shown to avoid crowding the figures. Ambient air pathogen detection rates are likely influenced by the sites being sampled in the different time periods, in addition to temporal variations in pathogen circulation in specific population subsets (Supplementary Figure 1).

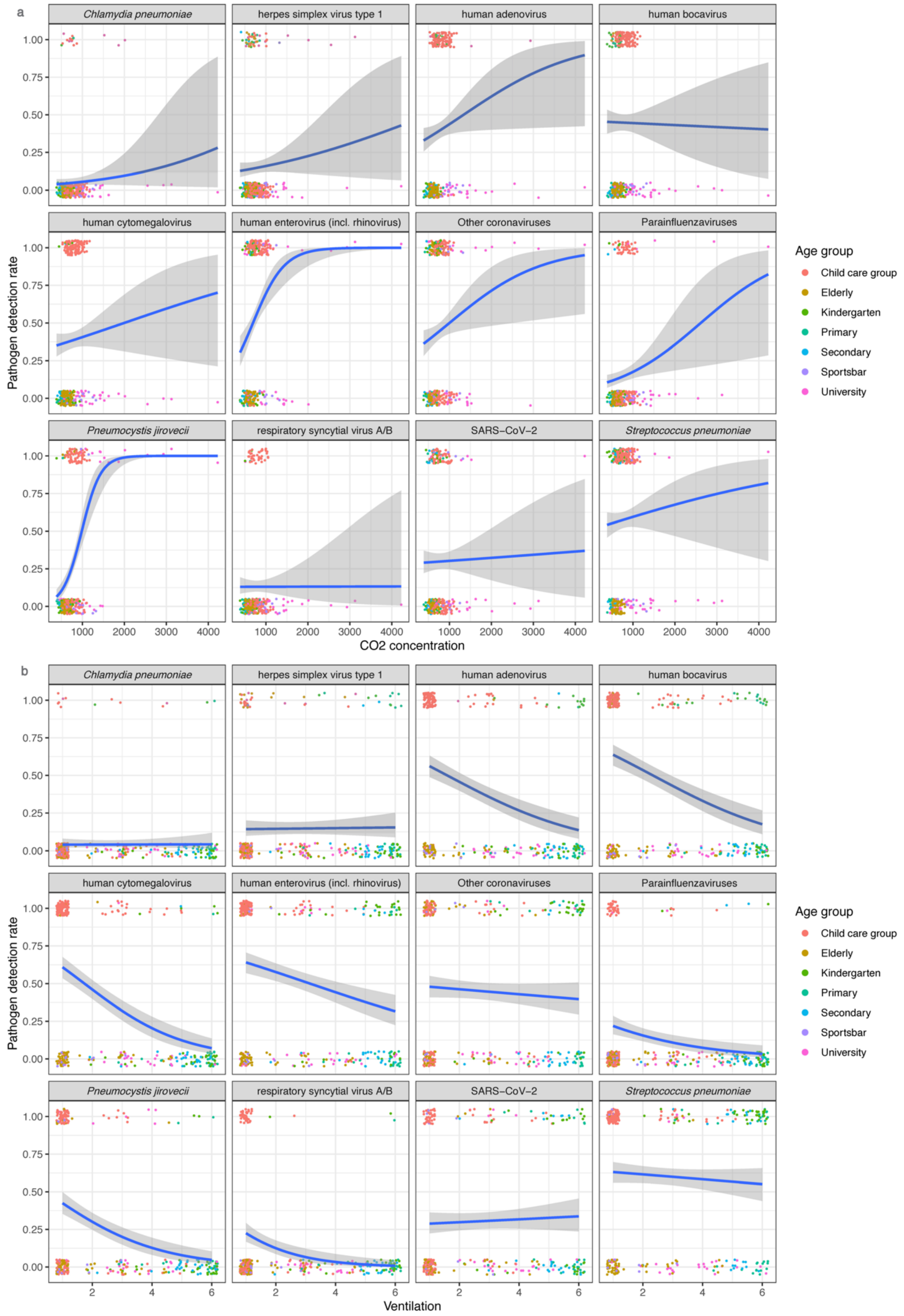

Supplementary Figure 3. Univariate associations between mean CO_2 or natural ventilation and the detection of (grouped) pathogens in indoor ambient air. *Pathogens, grouped as per Methods, which were positive in at least 10 air samples, are shown. Panel a) shows the univariate association between the mean CO_2 concentration and the probability of detecting a*

particular pathogen, with shaded areas representing 95% confidence intervals. Panel b) shows the univariate association between natural ventilation (as per a Likert scale) and the probability of detecting a pathogen, with shaded areas representing 95% confidence intervals. In most cases, the association is positive between CO_2 concentration and the risk of pathogen detection. The association is negative in most instances for natural ventilation. Counterintuitively, we don't see this expected association between natural ventilation and detection for SARS-CoV-2, even though confidence intervals are quite small across the range. This may indicate the presence of a strong confounder, such as stricter compliance with non-pharmaceutical interventions when incidence was high. Indeed, the association is not significant in a multivariate analysis (Supplementary Table 5).

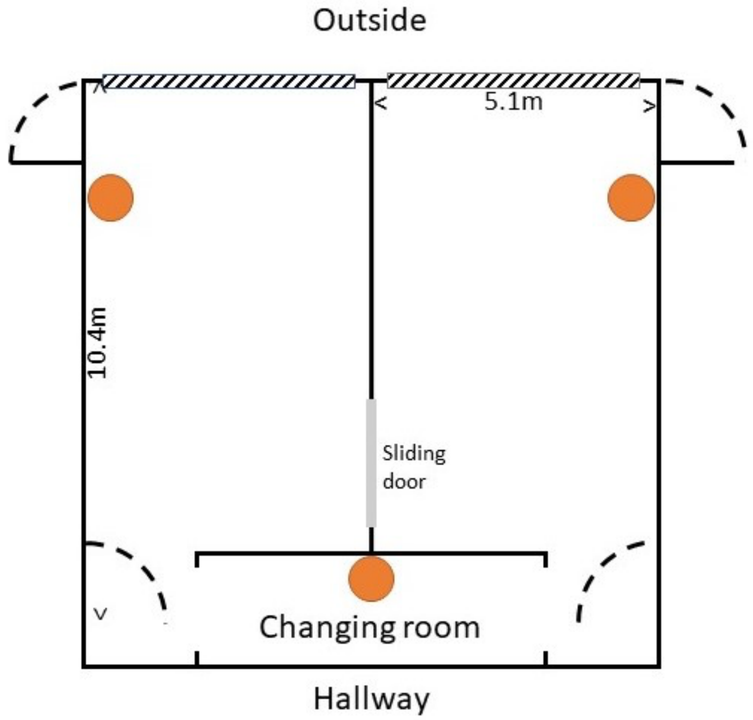

Supplementary Figure 4. Layout of two nursery sampling locations (locations 2 and 3) and placement of portable air filters. *The two main rooms were connected directly - through a sliding door which was most often open - and indirectly through the changing room. In both locations, one air filter was placed on the floor and two at 1m above the floor (orange). The third nursery sampling location had a shorter room length of 9.6m and equal width. This resulted in a volume of 150m^3, as opposed to 157m^3 in locations 2 and 3.*

Supplementary Tables

Site	Predominant age group	Room Volume (m^3)	Sampler height (m)	HVAC present	Portable air filters present	Aimed sampling duration	Aimed weekly frequency	Sampling period	Total N samples	Positivity rate sample	Mean N of detected pathogens
Nursery location 1	0y-3y	150,0	1.5	NO	NO	2h	3	15/11/21 – 27/4/22	60	98.93	7.00
Nursery location 2	0y-3y	157,5	1.5	NO	YES	2h	3	17/01/22 – 1/4/22	39	100.00	7.23
Nursery location 3	0y-3y	157,5	1.5	NO	YES	2h	3	07/2/22 – 1/4/22	24	100.00	7.75
Preschool cafeteria	3y-6y	372,0	1.7	NO	NO	2h	4	22/11/21 – 4/2/22	26	100.00	4.50
Preschool classroom	3y-6y	210,0	0.8	NO	NO	2h	1	08/12/21 – 2/2/22	4	100.00	4.75
Primary school cafeteria	6y-12y	930,0	1.7	NO	NO	1-2h	4	22/11/21 – 1/2/22	25	68.00	1.92
Primary school classroom	6y-12y	224,0	2	NO	NO	1h	1	08/12/21 – 2/2/22	4	100.00	4.75
Secondary school cafeteria	12y-18y	912,0	1.5	NO	NO	2h	4	23/11/21 – 28/3/22	24	79.17	1.29
University bar 1	18y-25y	200,0	2.5	NO	NO	2h	3	14/10/21 – 27/10/21	7	10.00	3.43

University bar 2	18y-25y	315,0	1.5	NO	NO	2h	3	18/10/21 – 10/11/21	7	100.00	3.29
University bar 3	18y-25y	198,0	1.2	YES	NO	2h	3	18/10/21 – 10/11/21	10	80.00	2.60
University cafeteria	18y-25y	7614,0	1.5	YES	NO	2h	4	18/10/21 – 29/10/21	10	80.00	1.60
University auditorium 1	18y-25y	324,0	1.3	NO	NO	2h	2	1/12/21 – 8/12/21	3	100.00	1.00
University auditorium 2	18y-25y	324,0	1.3	NO	NO	2h	2	4/11/21 – 25/11/21	13	38.46	0.46
University auditorium 3	18y-25y	324,0	0.6	NO	NO	2h	2	8/11/21 – 13/12/21	6	83.33	1.83
University auditorium 4	18y-25y	731,25	0.5	NO	NO	2h	1	14/12/21 – 14/12/21	1	100.00	1.00
Sports club bar	25-65y	324,0	0.6	NO	NO	2h	1	20/01/22 – 31/03/2022	10	90.00	2.00
Elderly care home 1	+65y	273,0	0.5	YES	NO	2h	4	9/12/21 – 1/02/22	19	78.95	1.32

Elderly care home 2	+65y	627,75	1	YES	NO	2h	4	6/12/21 – 4/02/22	35	77.14	1.71
Elderly care home 3a	+65y	600,0	1	YES	NO	2h	2	06/1/22 – 31/1/22	8	25.00	0.25
Elderly care home 3b	+65y	600,0	1	YES	NO	2h	2	06/1/22 – 24/1/22	6	33.33	0.33

Supplementary Table 1. The characteristics of the different sampling sites. *This table lists the different sampling sites. It shows their predominant age categories, room volume, the height at which the sampler was placed (rounded to 0.1m), the presence of a heating, ventilation and air conditioning (HVAC) system, and whether portable air filters were installed. It also lists the target sampling duration (accommodating site-specific schedules), the sampling period, the target sampling frequency and the total number of samples taken. The last columns list the crude positivity rate for any pathogen of all samples taken at a particular site and the average number of detected pathogens per sample. The aimed sampling duration was 2 hours, unless the site-specific schedule dictated otherwise (e.g. lunch time in schools). The following sites were in the same institution: nursery locations 1 to 3; pre-school cafeteria, pre-school classroom and primary*

school cafeteria; elderly care homes 3a and 3b. We focused on children and older people because of high incidence and morbidity from respiratory infections in these populations[11–13]*. For university auditoria, rooms where high* CO_2 *values were registered in the weeks prior to the start of the study were selected for inclusion. The nursery was adjacent to the University Hospital and hosts children of employees in the site. Samplers were placed off the ground and at maximum distance from attendants to avoid sampling resuspended aerosols or large exhaled particles as much as possible. One specific sampler was used in a particular location throughout the study. A sampler could be recommitted to a different location after interruption of sampling in one location.*

**Starting variables**	**Type**	**Missing data**	**Missing data handling**
Pathogen (grouped as per *Methods* description)	factor	0	
Predominant age group	factor	0	
Month of sampling	factor	0	
Sampling duration	numeric	0	
Mean CO_2	numeric	1/339	omit observation
Relative humidity	numeric	2/339	omit observations
Temperature	numeric	15/339	impute missing data
Natural ventilation	numeric	24/339	impute missing data
Air filtration	true/false	0	assume FALSE when missing
Mechanical ventilation	true/false	0	
Mean number of attendees	numeric	0	
Attendee density	numeric	0	
Mask wearing	numeric	64/339	impute missing data
Vocalization	numeric	73/339	impute missing data
Weekly COVID-19 incidence Leuven	numeric	0	

Supplementary Table 2. Missing host, behavioral and environmental values and measures taken in their presence. *A total of 2/339 observations were omitted due to missing data, and 0/337 because the data could not be imputed following the method described in Supplementary Methods.*

	a) Pathogen detection (all pathogens) in a logistic regression model		b) Pathogen concentration (all pathogens) in a linear regression model	
**Variable**	**p-value before backward elimination**	**Odds ratio and 95% CI before backward elimination**	**p-value before backward elimination**	**Coefficient and 95% CI before backward elimination**
Pathogen	< 0,0001		< 0,0001	
Predominant age group at the sampling site	< 0,0001		< 0,0001	
Month of sampling	0.0051		0.0046	
Sampling duration	0.2113	1.0022 (0.9988 – 1.0056)	0.2799	-0.0032 (-0.0090 – 0.0027)
Indoor CO_2 concentration	0.0013	1.0554 (1.0212 – 1.0907)	0.0017	-0.0800 (-0.1306 – -0.0295)
Relative humidity	0.1782	1.0114 (0.9949 – 1.0283)	0.8338	0.0027 (-0.0225 – 0.0278)
Indoor temperature	0.0986	1.0600 (0.9890 – 1.1361)	0.3823	0.0512 (-0.0653 – 0.168)
Natural ventilation	0.0193	0.8882 (0.8041 – 0.9811)	0.9068	0.0090 (-0.1431 – 0.1610)
Air filtration	0.1555	0.8181 (0.6203 – 1.0791)	0.0014	0.5400 (0.2042 - 0.8758)
Mechanical ventilation	0.3685	1.3382 (0.7121 – 2.5150)	0.1062	0.8895 (-0.2046 – 1.9837)
Number of attendees	0.4484	0.9981 (0.9929 – 1.0032)	0.8668	0.0008 (-0.0089 – 0.0105)
Attendee density	0.1151	3.5150 (0.7337 – 16.8387)	0.5593	0.8123 (-1.9533 – 3.5779)
Mask wearing	0.2427	1.0445 (0.9709 – 1.1237)	0.1934	-0.0842 (-0.2130 – 0.0445)
Vocalization	0.0157	0.8852 (0.8018 – 0.9772)	0.1379	-0.1208 (-0.2825 – 0.0410)
Weekly COVID-19 incidence Leuven	0.8112	1.0250 (0.8370 – 1.2553)	0.2918	0.1646 (-0.1457 – 0.4748)

Supplementary Table 3. Variable significance levels before backward elimination in generalized linear models. *Panel a) shows the two-sided p-values and odds ratios (with 95% CI) of the different variables, including inferred values, in a logistic regression model with*

pathogen detection as binary outcome. Panel b) shows the two-sided p-values and effect sizes % CI) of the different variables, including inferred values, in a linear regression model with pathogen concentration – measured by qPCR Ct value – as a numeric outcome. We used Chi squared tests to estimate p-values. 95% confidence intervals were computed as follows: coefficient estimate +/- standard error * 1.96.

a) Pathogen detection (all pathogens)			
**Remaining variables**	**p-value**	**Effect size (odd ratio and 95% CI)**	**Direction**
**Logistic regression model**			
Pathogen	<0.0001		
Age group	<0.0001		
Month	0.0024		
CO_2	0.0015	1.0894 (CI 1.0340 – 1.1478) per 100ppm increase	positive
Natural ventilation	0.0097	0.8797 (CI 0.7982 – 0.9696) per step increase	negative
**Mixed effects logistic regression model (random effect: sample ID)**			
Pathogen	<0.0001		
Age group	<0.0001		
Month	0.0025		
CO_2	0.0015	1.0894 (CI 1.0340 – 1.1478) per 100ppm increase	positive
Natural ventilation	0.0097	0.8797 (CI 0.7982 – 0.9696) per step increase	negative
**Generalized estimating equations model (grouped by sample ID)**			
Pathogen	<0.0001		
Age group	<0.0001		
Month	0.0016		
CO_2	0.0004	1.0900 (CI 1.0389 – 1.1436) per increase of 100ppm	positive
Natural ventilation	0.0044	0.8772 (CI 0.8017 – 0.9599) per step increase	negative
**b) Pathogen concentration (qPCR Ct of positive samples, all pathogens)**			
**Remaining variables**	**p-value**	**Effect size (change in Ct value and 95% CI)**	**Direction**
**Linear regression model**			
Pathogen	<0.0001		
Age group	<0.0001		
Month	0.0020		
CO_2	<0.0001	-0.0819 (CI -0.1229 – -0.0408) per increase of 100ppm	negative

Portable air filtration	0.0005	0.5813 (CI 0.2494 – 0.9131)	positive
**Mixed effects linear regression model (random effect: sample ID)**			
Pathogen	<0.0001		
Age group	<0.0001		
Month	0.0069		
CO_2	0.0003	-0.0799 (CI -0.1241 – -0.0357) per increase of 100ppm	negative
Portable air filtration	0.0020	0.5790 (CI 0.2052 – 0.9528)	positive

Supplementary Table 4 lists the pathogen, host, behavioural and environmental/building related factors significantly associated with indoor air bioaerosol load after backward elimination. *Panel a) shows the impact of variables on pathogen detection using a logistic regression model, mixed logistic regression model, and generalized estimating equations model (two-sided p-values, effect sizes with 95% CI). Panel b) shows the impact of variables on pathogen concentration using a linear regression model and mixed effects linear regression model (two-sided p-values, effect sizes with 95% CI). The significance levels and effect sizes of the input variables are almost identical across models (see also Table 1). We used the Wald test to estimate p-values in the generalized estimating equations model and the Chi squared test for the remainder of the models.*

**Pathogen**	**Variable**	**p-value**	**Odds ratio (95% CI)**
human adenovirus	NA	NA	NA
human bocavirus	Mean CO_2	0.0177	0.5720 (0.3604 – 0.9076)
Chlamydophila pneumoniae	NA	NA	NA
human cytomegalovirus	NA	NA	NA
human enterovirus (incl. rhinovirus)	Mean CO_2	0.0276	1.1398 (1.0146 – 1.2806)
herpes simplex virus type 1	NA	NA	NA
Other coronaviruses	Mean CO_2	0.0069	1.1595 (1.0415 – 1.2908)
human parainfluenzavirus 1	NA	NA	NA
Pneumocystis jirovecii	Mean CO_2	<0.0001	1.5639 (1.2663 – 1.9315)
Pneumocystis jirovecii	Mean natural ventilation	0.0317	0.6752 (0.4719 – 0.9662)
respiratory syncytial virus A/B	Mean natural ventilation	0.0070	0.4127 (0.2168 – 0.7856)
Streptococcus pneumoniae	Mean CO_2	0.0317	1.1106 (1.0092 – 1.2222)
SARS-CoV-2	NA	NA	NA

Supplementary Table 5 shows the influence of host, behavioural and environmental/building related factors on individual pathogen detection in a logistic regression model. *We built a model for each specific pathogen. Covariates were those independently associated with pathogen presence in previous models. We repeated backward elimination while always retaining the sampling month and dominant age group. The table lists the pathogen of interest and the retained variables after backward elimination (two-sided p-value <0,05). The last column shows the odds of detection per 100 ppm increase in* CO_2 *concentration or per stepwise increase in natural ventilation (Likert scale), and the corresponding 95% CI. P-values were assessed using the Chi squared method.*

Pathogen	Retained variable	p-value	Effect estimate (95% CI)
human adenovirus	Mean CO2	0.0158	-0,3297 (-0.5936 – -0.0658)
human bocavirus	Mean CO2	0.0001	-0.4368 (-0.6444 – -0.2291)
human bocavirus	Portable air filtration	0.0468	0.7002 (0.0159 – 1.3845)
Chlamydophila pneumoniae	NA	NA	NA
human cytomegalovirus	Mean CO2	0.0312	-0.2098 (-0.3984 – -0.0212)
human cytomegalovirus	Portable air filtration	0.0019	0.9644 (0.3700 – 1.5587)
human enterovirus (incl. rhinovirus)	NA	NA	NA
herpes simplex virus type 1	NA	NA	NA
Other coronaviruses	Portable air filtration	0.0019	2.0415 (0.7754 – 3.3075)
Parainfluenza viruses	NA	NA	NA
Pneumocystis jirovecii	NA	NA	NA
respiratory syncytial virus A/B	Mean CO2	0.0355	0.4742 (0.0493 – 0.8990)
Streptococcus pneumoniae	Mean CO2	0.0030	-0.1224 (-0.2023 – -0.0426)
Streptococcus pneumoniae	Portable air filtration	<0.0001	1.1064 (0.6082 – 1.6047)
SARS-CoV-2	NA	NA	NA

Supplementary Table 6 shows the influence of host, behavioural and environmental/building related factors on individual pathogen concentration (expressed as Ct value) in a linear regression model. *We built a model for each specific pathogen. Covariates were those independently associated with pathogen concentration in previous models. We repeated backward elimination while always retaining the sampling month and dominant age group. The table lists the pathogens of interest and the retained variables after backward elimination (two-sided p-value <0,05). The last column shows the estimated change in Ct value per 100 ppm increase in* CO_2 *concentration or stepwise increase in natural*

ventilation (Likert scale), and the 95% CI. P-values were assessed using the Chi squared method.

Site	HVAC/portable air filter type	Primary outdoor air fraction	Air transfer between zones	Recirculation within zone	Filter type (MERV)	Maximum volume displacement (m^3/h)	Draining volume (m^3)	Air changes per hour	On demand / continuous operation
Elderly care home 1	Stork-VDA200/4EC+WS-ventilator	NA	No	NA	NA	1000	273	3.7	On demand
Elderly care home 2	TRANE CCEC 9/4.5-9/4.5	~ CO_2 (ppm): 0% if < 400; 60% if 400-800; 100% if > 1200	No	Yes	8-9	45000	Unknown	Unknown	Continuous
Elderly care home 3a	CAIRplus 096.064IVBV	100 %	No	No	13-14	Unknown	425	Unknown	Continuous
Elderly care home 3b	CAIRplus 096.064IVBV	100 %	No	No	13-14	Unknown	425	Unknown	Continuous
University bar 3	Airoxy arok 2500mm fan	100 %	NA	NA	NA	1250	198	6.3	On demand

University cafeteria	CAIRplus 160.160IVBV	100%	No	NA	13-14	Unknown	7493	Unknown	~ CO_2 (ppm): Flow increases when >800 ppm
Child care group 2	Three Blue PURE 221 (Blueair®) portable air filters	0%	NA	100%	17	1,770	165	10.7	As per the sampling and filtration sequence in *Methods*
Child care group 3	Three Philips 3000i (Philips®) portable air filters	0%	NA	100%	17	999	165	6.1	As per the sampling and filtration sequence in *Methods*

Supplementary Table 7. HVAC/portable air filter characteristics. *The table shows the characteristics of HVAC systems or portable air filters for the sampling sites with such systems installed. Filter maintenance followed the manufacturer's prescription. For the two nursery locations equipped with portable air filters, the site and system specifics are shown. Air changes per hour are theoretical, as they are computed using the maximum volume displacement and the volume of the drained area.*

**Location**	**Portable air filtration phase**	**Change in Ct value (95% CI)**	**p-value**
Location 1	Wednesdays-Mondays	0.0155 (-0.4778 – 0.5088)	0.9506
	Fridays-Mondays	-0.0931 (-0.5864 – 0.4002)	0.7101
Location 2	Wednesdays-Mondays	1.220 (0.6514 – 1.7886)	<0.0001
	Fridays-Mondays	1.1340 (0.5654 – 1.7026)	0.0002

Location 3	Wednesdays-Mondays	0.3309 (-0.3169 – 0.9788)	0.3146
	Fridays-Mondays	1.0190 (0.3712 – 1.6669)	0.0026

Supplementary Table 8. The influence of air filtration on pathogen concentrations in an interventional comparison, assessed through a mixed effects linear regression model. *Repeated samples in different portable air filtration phases were used as inputs (see Methods). The change in mean Ct value of all positive respiratory pathogens against the baseline value on Mondays was the model outcome (see also Figure 2). Confidence intervals were calculated using the confint command in R, p-values were obtained using the Kenward-Roger approximation of the t-distribution (pbkrtest package in R).*

**a) Pathogen detection (all pathogens) in a logistic regression model with alternative SARS-CoV-2 results**			
**Remaining variables**	**p-value**	**Effect size (odd ratio and 95% CI)**	**Direction**
Pathogen	<0.0001		
Age group	<0.0001		
Month	0,0032		
CO_2	0,0007	1.1029 (CI 1.0429 – 1.1664) per increase of 100ppm	positive
Natural ventilation	0,0129	0.8763 (CI 0.7895 – 0.9727) per step increase	negative
**b) Pathogen concentration (qPCR Ct of positive samples, all pathogens) in a linear regression model with alternative SARS-CoV-2 results**			
**Remaining variables**	**p-value**	**Effect size (change in Ct value and 95% CI)**	**Direction**
Pathogen	<0.0001		
Age group	<0.0001		
Month	0,0022		
CO_2	<0.0001	-0.0888 (CI -0.1325 – -0.0451) per increase of 100ppm	negative
Portable air filtration	0,0012	0.5623 (CI 0.2196 – 0.9050)	positive

Supplementary Table 9 uses the respiratory panel qPCR results for SARS-CoV-2 instead of the TaqPath results as input, and lists the pathogen, host, behavioural and

environmental/building related factors significantly associated with indoor air bioaerosol load after backward elimination. *As in the main analysis (see Table 1), the influence on pathogen detection was assessed using a logistic regression model (panel a), while the influence on pathogen concentration was assessed using a linear regression model (panel b). In both, two-sided p-values were estimated using the Chi squared method. Odds ratio's and 95% CI are also shown. P-values and effect sized are nearly identical to the main analysis.*

		Taqpath SARS-CoV-2 qPCR							
		ORF1ab		N gene		S gene		Any gene	
		Pos	Neg	Pos	Neg	Pos	Neg	Pos	Neg
Respiratory panel (ORF1ab)	Pos	7	2	7	2	4	5	7	2
	Neg	70	224	78	216	23	271	83	211

Supplementary Table 10. The influence of the qPCR panel on SARS-CoV-2 detection in ambient air samples. *This table compares the results (positive or negative) of the in-house respiratory panel SARS-CoV-2 qPCR (targeting ORF1ab) and the TaqPath SARS-CoV-2 qPCR assay. We stratified the result of the latter by its genetic targets (ORF1ab, N and S) and all targets aggregated (Any gene). N air samples = 303. Sensitivity differed significantly when comparing the respiratory panel either to the ORF1ab target only or to all targets in the Taqpath SARS-CoV-2 qPCR (McNemar test, two-sided p-values <0.0001 for both comparisons, no correction for multiple testing). See also Supplementary Methods.*

**Sample**	**Respiratory panel SARS-CoV-2 qPCR** (ORF1ab directed)	**In-house SARS-CoV-2 qPCR** (E-gene directed)
1) QCMD CVOP20S		
Sample 1 (4.3 log/ml SARS-CoV-2)	31.3	26.1
Sample 2 (*Human coronavirus NL63*)	neg	neg
Sample 3 (3.3 log/ml SARS-CoV-2)	34.3	29.8
Sample 4 (*Human coronavirus OC43*)	neg	neg
Sample 5 (negative control)	neg	neg
Sample 6 (4.3 log/ml SARS-CoV-2)	31.5	26.2
Sample 7 (5.3 log/ml SARS-CoV-2)	28.4	23.8
Sample 8 (2.3 log/ml SARS-CoV-2)	37.8	33.5
2) Qnostics standardized samples		
Qnostics 2000 c/ml	33.7	30.5
Qnostics 1000 c/ml	34.9	31.5
Qnostics 500 c/ml	36.3	32.5
Qnostics 250 c/ml	neg	32.7

3) Strong positive SARS-CoV-2 clinical sample (Wuhan strain)		
10^{-1} dilution	23.7	19.0
10-2 dilution	27.1	22.2
10^{-3} dilution	30.5	25.7
10^{-4} dilution	33.9	28.8
10^{-5} dilution	36.1	32.3
Negative control	neg	neg
4) Stored inactivated culture (Wuhan strain)		
10^{-4} dilution	27.6	23.7
10^{-5} dilution	30.3	26.7
10^{-6} dilution	33.4	30.0
10^{-7} dilution	35.8	33.3
Negative control	neg	neg

Supplementary Table 11. Validation experiments of respiratory panel SARS-CoV-2 qPCR. *This table lists 4 validation experiments comparing qPCR results (positive or negative) and Ct values of two tests: the in-house respiratory panel qPCR directed against the ORF1ab gene of SARS-CoV-1/2 and an in-house single plex qPCR directed against the E-gene of SARS-CoV-2 (see also Supplementary Methods). Validation experiment 1) was a blinded QCMD series (samples 1 to 6), used in the Coronavirus disease (COVID-19) outbreak preparedness EQA pilot study. The content of the unblinded samples is shown between brackets. Experiment 2) was run on a series of commercially sourced samples (Qnostics®) with a known number of gene copies per milliliter. Experiment 3) was run on a dilution series of a strongly positive patient sample with confirmed SARS-CoV-2 infection (Wuhan strain). Experiment 4) was conducted on a dilution series of a stored, heat-inactivates SARS-CoV-2 culture of the sample patient sample (Wuhan strain), which was used in a national effort to standardize SARS-CoV-2 diagnosis and reporting by Belgian clinical laboratories*[6]*.*

Multiplex qPCR	Pathogen	Primer/probe	Primer/probe sequence (5’ à 3’)	Concentration (nM)	Target gene	Amplicon size (bp)	Ct cut-off positive versus negative	Validation since last change to Multiplex qPCR
1	Influenza A virus	Forward primer	TCTCATGGAATGGCTAAAGACAAG	300	Matrix protein gene	116	NA	QCMD Respiratory I EQA Programme
		Reverse primer	CAAAGCGTCTACGCTGCAGT	300				
		Probe	FAM-TTCACGCTCACCGTGC-MGB	200				
	Influenza B virus	Forward primer	AATACGGTGGATTAAACAAAAGCA	300	Hemagluttinin gene	170	NA	QCMD Respiratory I EQA Programme
		Reverse primer	ACCAGCAATAGCTCCGAAGAA	300				
		Probe	NED-TATATTTGGTTCCATTGGC-MGB	200				
	Phocine Distemper Virus (RNA Internal control)	Forward primer	GGTGGGTGCCTTTTACAAGAAC ([14])	250	Hemagluttinin gene	83	NA	NA
		Reverse primer	ATCTTCTTTCCTCAACCTCGTCC ([14])	250				
		Probe	VIC-ATGCAAGGGCCAATT-MGB ([14])	100				
2	Respiratory syncytial virus A/B	Forward primer	AATACAGCCAAATCTAACCAACTTTACA ([15])	300	RNA polymerase gene	94	NA	QCMD Respiratory I EQA Programme
		Reverse primer	GCCAAGGAAGCATGCAATAAA ([15])	300				
		Probe A	FAM-TGATGTGCTATTGTGCACTA-MGB ([15])	100				
		Probe B	FAM-CACTATTCCTTACTAAAGATGTC-MGB ([15])	100				
	Human metapneumovirus	Forward primer	GCYGTYAGCTTCAGTCAATTCAA	500	Fusion protein gene	75	NA	QCMD Respiratory II EQA Programme
		Reverse primer	TGTTATYCCWGCATTGTCTGA	500				
		Probe	NED-CTAAATGTTGTGCGGCAAT-MGB	200				
3	Parainfluenza virus 1	Forward primer	CCTTCATTATCAATTGGTGATGCA	500	Hemagglutinin-	180	NA	QCMD Respirator

		Reverse primer	CCTGTTGTCGTTGATGTCATAGGT	500	neuraminidase gene			y II EQA Programme
		Probe	FAM-TCAAACTTAATCACTCAAGGAT-MGB	100				
	Parainfluenza virus 2	Forward primer	CATTTACCTAAGTGATGGAATCAATCG	500	Hemagglutinin - neuraminidase gene	141	36.5	IQC-1
		Reverse primer	GCAAGTCTCAGTTCAGCTAGATCAGT	500				
		Probe	NED-AAAGCTGTTCAGTCACTG-MGB	150				
4	Parainfluenza virus 3	Forward primer	CAAAGTTGATGAAAGATCAGATTATGC	500	Hemagglutinin - neuraminidase gene	169	NA	IQC-1
		Reverse primer	GTAGTATATCCCTGGTCCAACAGATG	500				
		Probe	FAM-CAATCTCRACAACAAGATT-MGB	100				
	Parainfluenza virus 4	Forward primer	GGAGCAAAAGAYTCATACACAATAACTTACT	500	Hemagglutinin - neuraminidase gene	116	NA	IQC-1
		Reverse primer	CTAGGAATTAARATTTCACAATCTTCAGAA	500				
		Probe	NED-AAATGCACTCTGTATAAGTC-MGB	150				

**Multiplex PCR**	**Pathogen**	**Primer/probe**	**Primer/probe sequence (5’ à 3’)**	**Concentration (nM)**	**Target gene**	**Amplicon size (bp)**	**Ct cut-off positive versus negative**	**Validation since last change to Multiplex qPCR**
5	Human adenovirus	Forward primer	TYGARGTGGAYCCCATGGAYGAG	800	Hexon gene	112	39.0	QCMD Respiratory II EQA Programme
		Reverse primer	CGCAGGTAGACBGCYTCRATGA	800				
		Probe 1	FAM-ACGTCGAAGACTTC-MGB	100				
		Probe 2	FAM-ACGTCGAAAACTTC-MGB	100				
		Probe 3	FAM-CACGTCAAAGACTTC-MGB	100				
	Human enterovirus (inc. rhinovirus)	Forward primer 1	CAWGGTGYGAAGAGYCTATTGAGCT	500	Polyprotein gene	150-154	NA	QCMD Respiratory II EQA Programme
		Forward primer 2	GTGTGAAGASCCSMGTGYGCT	500				

		Reverse primer	GAAACACGGACACCCAAAGTAGT	500				
		Probe	VIC-TCCGGCCCCTGAATGYGGCTAA-TAMRA	200				
6	Human cytomegalovirus	Forward primer	CGTAACGTGGACCTGACGTTT	250	Major capsid protein gene	148	37.0	IQC-1
		Reverse primer	CACGGTCCCGGTTTAGCA	250				
		Probe	FAM-TATCTGCCCGAGGATCGCGGTTACA-TAMRA	200				
	Human parechovirus	Forward primer	CASWWGCCTCTGGGSCCAAAAG ([16])	500	Polyprotein gene	189	36.0	QCMD Parechovirus RNA EQA Programme
		Reverse primer	GGCCCCWGRTCAGATCCAYAGT ([16])	500				
		Probe	Cy5-CCTRYGGGTACCTYCWGGGCATCCTTC-BHQ3 ([16])	200				
	Phocine Herpesvirus-1 (DNA internal control)	Forward primer	GGGCGAATCACAGATTGAATC ([17])	100	Glycoprotein B gene	89	NA	NA
		Reverse primer	GCGGTTCCAAACGTACCAA ([17])	100				
		Probe	VIC-TCCGCCACCATCTG-MGB	100				
7	*Human coronavirus NL63*	Forward primer	CAGGGCTGACAAGCCTTCTCA ([18])	500	Nucleocapsid protein gene	144	35.0	QCMD Respiratory II EQA Programme
		Reverse primer	GCATCAACACCATTCTGAACAAGA ([18])	500				
		Probe	FAM-CGTTGGAAGCGTGTTCCTACCAGAGAGG-BHQ-1 ([18])	200				
	Human coronavirus 229E	Forward primer	TGGAAGTGCAGGTGTTGTGGC ([18])	500	Nucleocapsid protein gene	99	34.5	IQC-1
		Reverse primer	TGACTATCAAACAGCATAGCAGCTG ([18])	500				
		Probe	Cy5-CCACAATTTGCTGAGCTTGTGCCGTC-BHQ-3 ([18])	200				
	Human coronavirus OC43	Forward primer	CGATGAGGCTATTCCGACTAGGT ([19])	500	Nucleocapsid protein gene	75	35.5	QCMD Respiratory II EQA Programme
		Reverse primer	CCTTCCTGAGCCTTCAATATAGTAACC ([19])	500				
		Probe	VIC-TCCGCCTGGCACGGTACTCCCT-TAMRA ([19])	200				

Multiplex PCR	Pathogen	Primer/probe	Primer/probe sequence (5’ à 3’)	Concentration (nM)	Target gene	Amplicon size (bp)	Ct cut-off positive versus negative	Validation since last change to Multiplex qPCR
8	Herpes simplex virus 1 & 2	Forward primer	AACCTGGGRTTCCTGATGCA	250	Nonfunctional glycoprotein D gene (US6)	84		HSV-1: IQC-1<br>HSV-2: IQC-1
		Reverse primer	CTCCGTCCAGTCGTTTATCTTCAC	250				
		Probe HSV-1	FAM-TTTGAGACCGCCGGCACGTAC-BHQ-1	100			NA	
		Probe HSV-2	Cy5-CCTTCGAGACCGCGGGTACGTA-BHQ-3	100			35.0	
	Enterovirus D68	Forward primer	TGGCGGCCTACTCATGG ([20])	500	Polyprotein gene	64	NA	QCMD Respiratory II EQA Programme
		Reverse primer	AATAGACTCTTCACACCTTGTTCATGT ([20])	500				
		Probe	NED-AAAACCATGAGACGCT-MGB ([20])	200				
	SARS-CoV-1 + SARS-CoV-2	Forward primer	CWGGCATACCWAAGGACATGACCTA	500	ORF1ab polyprotein gene	147	38.0	IQC-1
		Reverse primer	CKACATCRAAGCCAATCCA	500				
		Probe	VIC-TTTATCACCCGCGAAGAA-MGB	200				
9	*Mycoplasma pneumoniae*	Forward primer	AGGCTTCAAGTGGACAAAGTGAC	250	Adhesin P1 gene	76	NA	QCMD Respiratory III EQA Programme
		Reverse primer	GATTGTYCCTGCTGGYCCAT	250				
		Probe	FAM-ACCACACCAAGTTCA-MGB	200				
	Human coronavirus HKU1	Forward primer	CACTTCTATTCCCTCCGATGTTTC ([18])	500	Nucleocapsid protein gene	129	32.5	IQC-1
		Reverse primer	TTAGAAGCAGACCTTCCTGAGCC ([18])	500				
		Probe	Cy5-CGCCTGGTACGATTTTGCCTCAAGGCT-BHQ-3 ([18])	200				

10	*Pneumocystis jirovecii*	Forward primer	GCACTGAATATCTCGAGGGAGTATG	250	Large subunit ribosomal RNA gene	145	NA	QCMD Pneumocystis jirovecii pneumonia (PCP) DNA EQA Programme
		Reverse primer	TTGGGAGCTTTAATTACTGTTCTGG	250				
		Probe	FAM-TGTTTCCCTTTCGACTATC-MGB	200				
	Coxiella burnetii	Forward primer	CGATAGCCCGATAAGCATCAAC ([21])	250	IS1111 transposase gene	88	36.7	IQC-1
		Reverse primer	TGCATTCGTATATCCGGCATC ([21])	250				
		Probe	NED-TCATCAAGGCACCAATG-MGB ([21])	200				
	MERS-CoV	Forward primer	GCAACGCGCGATTCAGTT ([22])	500	Envelope gene (upE)	92	35.5	QCMD MERS Coronavirus EQA Programme
		Reverse primer	GCCTCTACACGGGACCCATA ([22])	500				
		Probe	Cy5-CTCTTCACATAATCGCCCCGAGCTCG-BHQ-3 ([22])	200				

**Multiplex PCR**	**Pathogen**	**Primer/probe**	**Primer/probe sequence (5’ à 3’)**	**Concentration (nM)**	**Target gene**	**Amplicon size (bp)**	**Ct cut-off positive versus negative**	**Validation since last change to Multiplex qPCR**
11	*Chlamydia pneumoniae*	Forward primer	CATCCGTGTCGGAGCTAACGT ([23])	250	16S rRNA gene	184	34.5	QCMD Respiratory III and Chlamydophila pneumoniae EQA Programme
		Reverse primer	TGCGGAAAGCTGTATTTCTACAGTT ([23])	250				
		Probe	FAM-ATGCCGCCTGAGGAGTACACTCGC-BHQ1 ([23])	200				
	Chlamydia psittaci	Forward primer	GCCATCATGCTTGTTTCGTTT ([24])	250	Inclusion membrane protein A gene	74	40.0	QCMD Chlamydia psittaci EQA Programme
		Reverse primer	CGGCGTGCCACTTGAGA ([24])	250				
		Probe	VIC-TTGTCATTATGGTGATTCAGGA-MGB ([24])	200				

	Streptococcus pneumoniae	Forward primer	ACGCAATCTAGCAGATGAAGCA ([25])	250	Autolysin gene (lytA)	75	36.0	QCMD Respiratory III EQA Programme
		Reverse primer	TCGTGCGTTTTAATTCCAGCT ([25])	250				
		Probe	Cy5-TGCCGAAAACGCTTGATACAGGGAG-BHQ3 ([25])	200				
12	*Legionella pneumophila*	Forward primer	TCCGGAAGCAATGGCTAAAG	250	Macrophage infectivity potentiator gene	69	35.5	QCMD Respiratory III EQA Programme
		Reverse primer	TGCTGTTCGGTTAAAGCCAAT	250				
		Probe	FAM-CATGCAAGACGCTATGAGTGGCGCT-TAMRA	200				
	Human bocavirus	Forward primer	GCACAGCCACGTGACGAA ([26])	250	Nonstructural protein 1 gene	76	34.5	IQC-1
		Reverse primer	TGGACTCCCTTTTCTTTTGTAGGA ([26])	250				
		Probe	Cy5-TGAGCTCAGGGAATATGAAAGACAAGCATCG-BHQ-3 ([26])	200				

Supplementary Table 12. List of primer and probe sequences and final concentrations used in the 12 multiplex PCRs for the respiratory panel, targeting a total of 29 individual pathogens. *Ambiguity codes: R: A or G; Y: C or T; W: A or T; S: C or G; K: G or T. Cut-off Ct values are based on the reexamination of samples with possible non-specific amplification and the results from those reruns (see Supplementary Methods). NA signifies no possible non-specific amplification has taken place so that no cutoff Ct has had to be determined. The last column highlights the highest level of validation to which each pathogen specific qPCR was subjected in the past 5 years. Validation methods listed from lowest to highest stringency levels are: IQC-1 = Internal non-blinded quality control; QCMD = external quality control on Quality Control for Molecular Diagnostics (QCMD) samples*[3].

Pathogen	N positive	N possibly non-specific	N retested	N retested positive	Decision possible non-specific
influenza A virus	4	0	0	NA	NA
influenza B virus	0	0	0	NA	NA
respiratory syncytial virus A/B	43	0	0	NA	NA
human metapneumovirus	3	0	0	NA	NA
human parainfluenza virus 1	0	0	0	NA	NA
human parainfluenza virus 2	3	3	3	0	Classify as negative
human parainfluenza virus 3	43	0	0	NA	NA
human parainfluenza virus 4	9	0	0	NA	NA
human adenovirus	134	9	5	3	Classify as positive
human enterovirus (incl. rhinovirus)	182	0	0	NA	NA
human cytomegalovirus	131	1	1	0	Classify as negative
human parechovirus	5	5	5	0	Classify as negative
Human coronavirus NL63	13	13	5	0	Classify as negative
Human coronavirus 229E	37	28	5	4	Classify as positive
Human coronavirus OC43	32	21	5	2	Classify as negative
herpes simplex virus type 1	50	0	0	NA	NA
herpes simplex virus type 2	0	0	0	NA	NA
enterovirus D68	6	0	0	NA	NA
SARS-CoV-2	10	2	0	NA	NA

Mycoplasma pneumoniae	1	0	0	NA	NA
Human coronavirus HKU1	98	85	5	5	Classify as positive
Pneumocystis jirovecii	100	0	0	NA	NA
Coxiella burnetii	4	3	3	1	Classify as negative
MERS-CoV	3	3	3	0	Classify as negative
Chlamydia pneumoniae	23	23	5	1	Classify as negative
Chlamydia psittaci	1	1	1	0	Classify as negative
Streptococcus pneumoniae	195	0	0	NA	NA
Legionella pneumophila	0	0	0	NA	NA
human bocavirus	152	29	5	4	Classify as positive

Supplementary Table 13. *Results of respiratory panels with Ct values compatible with possible non-specific amplification. Ct values compatible with possible non-specific amplification according to clinical cutoffs for respiratory samples (see Supplementary Table 12), and the results of repeated testing using qPCR with fresh primer probe mix. A total of 226 Ct values were possibly compatible with non-specific amplification. After the initial rerun of up to 5 samples with the lowest Ct values per pathogen (less than 5 samples were run per pathogen only when less than 5 samples had possible non-specific amplification for that pathogen). If at least 60% of samples returned positive for, we retained the initial result. If less than 60% of samples returned positive, all samples with possible non-specific amplification for that pathogen were reclassified as negative.*

Supplementary references

1. Belac | FPS Economy. https://economie.fgov.be/belac.
2. Rabenau, H. F. *et al.* Verification and validation of diagnostic laboratory tests in clinical virology. *Journal of Clinical Virology* **40**, 93–98 (2007).
3. QCMD. https://www.qcmd.org/.
4. Beuselinck, K., Bruyninckx, K. & Lagrou, K. Evaluation of Panther Fusion respiratory panel assays (Hologic) on respiratory samples. in *21 ESCV European society for clinical virology* 145–145 (2018).
5. Matheeussen, V. *et al.* International external quality assessment for SARSCoV-2 molecular detection and survey on clinical laboratory preparedness during the COVID-19 pandemic. *Eurosurveillance* **25**, 2001223 (2020).
6. Cuypers, L. *et al.* Nationwide Harmonization Effort for Semi-Quantitative Reporting of SARS-CoV-2 PCR Test Results in Belgium. *Viruses 2022, Vol. 14, Page 1294* **14**, 1294 (2022).
7. Cuypers, L. *et al.* Comprehensive immunovirological and environmental screening reveals risk factors for fatal COVID-19 during post-vaccination nursing home outbreaks. doi:10.21203/rs.3.rs-1479515/v1.
8. Horve, P. F., Dietz, L., Northcutt, D., Stenson, J. & van den Wymelenberg, K. Evaluation of a bioaerosol sampler for indoor environmental surveillance of Severe Acute Respiratory Syndrome Coronavirus 2. *PLoS One* **16**, e0257689 (2021).
9. Indoor air quality probe for CO2, temperature, humidity and absolute pressure | Testo international. https://www.testo.com/en/iaq-probe-to-assess-indoor-air-quality-co2-humi/p/0632-1535.
10. ERS CO2 – en. https://www.elsys.se/en/ers-co2/.
11. Ramaekers, K. *et al.* Prevalence and seasonality of six respiratory viruses during five consecutive epidemic seasons in Belgium. *J Clin Virol* **94**, 72–78 (2017).
12. Putri, W. C. W. S., Muscatello, D. J., Stockwell, M. S. & Newall, A. T. Economic burden of seasonal influenza in the United States. *Vaccine* **36**, 3960–3966 (2018).
13. Fendrick, A. M., Monto, A. S., Nightengale, B. & Sarnes, M. The economic burden of non-influenza-related viral respiratory tract infection in the United States. *Arch Intern Med* **163**, 487–494 (2003).
14. Clancy, A., Crowley, B., Niesters, H. & Herra, C. The development of a qualitative real-time RT-PCR assay for the detection of hepatitis C virus. *European Journal of Clinical Microbiology and Infectious Diseases* **27**, 1177–1182 (2008).
15. Kuypers, J., Wright, N. & Morrow, R. Evaluation of quantitative and type-specific real-time RT-PCR assays for detection of respiratory syncytial virus in respiratory specimens from children. *Journal of Clinical Virology* **31**, 123–129 (2004).
16. Nix, W. A. *et al.* Detection of all known parechoviruses by real-time PCR. *J Clin Microbiol* **46**, 2519–2524 (2008).

17. Niesters, H. G. M. Molecular and diagnostic clinical virology in real time. *Clinical Microbiology and Infection* **10**, 5–11 (2004).

18. Tiveljung-Lindell, A. *et al.* Development and implementation of a molecular diagnostic platform for daily rapid detection of 15 respiratory viruses. *J Med Virol* **81**, 167–175 (2009).

19. van Elden, L. J. R. *et al.* Frequent Detection of Human Coronaviruses in Clinical Specimens from Patients with Respiratory Tract Infection by Use of a Novel Real-Time Reverse-Transcriptase Polymerase Chain Reaction. *J Infect Dis* **189**, 652–657 (2004).

20. Piralla, A., Girello, A., Premoli, M. & Baldanti, F. A new real-time reverse transcription-PCR assay for detection of human enterovirus 68 in respiratory samples. *J Clin Microbiol* **53**, 1725–1726 (2015).

21. Panning, M. *et al.* High throughput detection of Coxiella burnetii by real-time PCR with internal control system and automated DNA preparation. *BMC Microbiol* **8**, 77 (2008).

22. Corman, V. M. *et al.* Detection of a novel human coronavirus by real-time reverse-transcription polymerase chain reaction. *Eurosurveillance* **17**, (2012).

23. Hardick, J. *et al.* Real-time PCR for Chlamydia pneumoniae utilizing the Roche Lightcycler and a 16S rRNA gene target. *Journal of Molecular Diagnostics* **6**, 132–136 (2004).

24. Ménard, A. *et al.* Development of a real-time PCR for the detection of Chlamydia psittaci [2]. *Journal of Medical Microbiology* vol. 55 471–473 Preprint at https://doi.org/10.1099/jmm.0.46335-0 (2006).

25. Carvalho, M. D. G. S. *et al.* Evaluation and improvement of real-time PCR assays targeting lytA, ply, and psaA genes for detection of pneumococcal DNA. *J Clin Microbiol* **45**, 2460–2466 (2007).

26. Neske, F. *et al.* Real-time PCR for diagnosis of human bocavirus infections and phylogenetic analysis. *J Clin Microbiol* **45**, 2116–2122 (2007).
