# Supplementary material for: Indoor air surveillance and factors associated with respiratory pathogen detection in community settings in Belgium
Source: Nat Commun. 2023 Mar 11;14:1332. doi: 10.1038/s41467-023-36986-z (PMC10005919; doi:10.1038/s41467-023-36986-z)
Supplement: Supplementary file 5 — Reporting Summary [file 41467_2023_36986_MOESM5_ESM.pdf]

nature portfolio

Corresponding author(s): Joren Raymenants

Last updated by author(s): Feb 10, 2023

Reporting Summary

Nature Portfolio wishes to improve the reproducibility of the work that we publish. This form provides structure for consistency and transparency in reporting. For further information on Nature Portfolio policies, see our Editorial Policies and the Editorial Policy Checklist.

Statistics

For all statistical analyses, confirm that the following items are present in the figure legend, table legend, main text, or Methods section.

n/a	Confirmed	
☐	☒	The exact sample size (*n*) for each experimental group/condition, given as a discrete number and unit of measurement
☐	☒	A statement on whether measurements were taken from distinct samples or whether the same sample was measured repeatedly
☐	☒	The statistical test(s) used AND whether they are one- or two-sided<br>*Only common tests should be described solely by name; describe more complex techniques in the Methods section.*
☐	☒	A description of all covariates tested
☐	☒	A description of any assumptions or corrections, such as tests of normality and adjustment for multiple comparisons
☐	☒	A full description of the statistical parameters including central tendency (e.g. means) or other basic estimates (e.g. regression coefficient) AND variation (e.g. standard deviation) or associated estimates of uncertainty (e.g. confidence intervals)
☐	☒	For null hypothesis testing, the test statistic (e.g. *F*, *t*, *r*) with confidence intervals, effect sizes, degrees of freedom and *P* value noted<br>*Give P values as exact values whenever suitable.*
☒	☐	For Bayesian analysis, information on the choice of priors and Markov chain Monte Carlo settings
☐	☒	For hierarchical and complex designs, identification of the appropriate level for tests and full reporting of outcomes
☐	☒	Estimates of effect sizes (e.g. Cohen's *d*, Pearson's *r*), indicating how they were calculated

Our web collection on statistics for biologists contains articles on many of the points above.

Software and code

Policy information about availability of computer code

Data collection	Manual data collection took place on paper, after which it was inputted in Excel version 16.68 (Microsoft®). Continuous measurements of ambient air parameters were collected on the web based platform (Grafana, Grafana Labs®) and downloaded as CSV files. Laboratory test results were downloaded from the UZ Leuven laboratory information system as CSV files.
Data analysis	Data analysis was performed either using R script in R versions 4.0.2/4.0.3/4.1.1 or python script in python version 3.8 specifically written for this study. The analyses performed as part of this research paper are publicly available on https://github.com/jraymenants/envir-air-sampling.

For manuscripts utilizing custom algorithms or software that are central to the research but not yet described in published literature, software must be made available to editors and reviewers. We strongly encourage code deposition in a community repository (e.g. GitHub). See the Nature Portfolio guidelines for submitting code & software for further information.

Data

Policy information about availability of data

All manuscripts must include a data availability statement. This statement should provide the following information, where applicable:

- Accession codes, unique identifiers, or web links for publicly available datasets
- A description of any restrictions on data availability
- For clinical datasets or third party data, please ensure that the statement adheres to our policy

Supplementary Data 1 contains the Supplementary methods, Supplementary Figures and Supplementary Tables. All data related to pathogen presence in

environmental air are added in Supplementary Data 2 and 3. Supplementary Data 2 contains grouped pathogens, while Supplementary Data 3 contains ungrouped pathogens.

Human research participants

Policy information about studies involving human research participants and Sex and Gender in Research.

Reporting on sex and gender	NA
Population characteristics	NA
Recruitment	NA
Ethics oversight	The study received approval from the Ethics Committee Research UZ / KU Leuven (S66518, B3222022000873). No informed consent was required from occupants of the sampled environments. Sampling took place after the management of each institution had agreed to take part in the study.

Note that full information on the approval of the study protocol must also be provided in the manuscript.

Field-specific reporting

Please select the one below that is the best fit for your research. If you are not sure, read the appropriate sections before making your selection.

☐ Life sciences ☐ Behavioural & social sciences ☒ Ecological, evolutionary & environmental sciences

For a reference copy of the document with all sections, see nature.com/documents/nr-reporting-summary-flat.pdf

Ecological, evolutionary & environmental sciences study design

All studies must disclose on these points even when the disclosure is negative.

Study description	The study assessed the presence and concentration of a series of 29 respiratory pathogens in the ambient air of community settings in Leuven, Belgium, over a 7 Month period. We provide a descriptive analysis of the occurrence of pathogens.<br>The study also assessed the influence of pathogen, host, behavioural and environmental/building related factors on pathogen detection and concentration. For this analysis, we used different types of multivariate models (GLM, GEE, GLMM) depending on the outcome (binary for detection and numerical for concentration).<br>In an interventional sub-study, we assessed the influence of air filtration on the number and concentration of pathogens in a childcare setting (which had a high burden of pathogens). We used Cochran Q test to assess the influence of air filtration on pathogen occurrence. We used mixed effects linear regression (with pathogen and week as random factors) to assess the influence on concentration.<br>In an exploratory analysis, we investigated whether the pathogens found in ambient air samples from community settings corresponded to the pathogens found in patients with severe respiratory infections in the same region and period. We therefore retrieved the results of the same, 29 pathogen qPCR panel, performed on respiratory samples of patients at University Hospitals Leuven.
Research sample	We selected a convenience sample of community settings in and around the city of Leuven, Belgium. Sampling sites covered different predominant age groups: nursery (0-3y), preschool (3-6y), primary school (6-12y), secondary school (12-18y), adults (18+y) and older nursing homes (65+). We focused on children and older people because of high morbidity from respiratory infections in these populations.<br>De data on behavioural/invoronmental factors (CO2, temperature, occupancy, vocalisation etc - see source-data.xlsx and lower for the full range of cofactors) was collected either on paper and excel, or within a web application (for continuous measurement of environmental samples). Laboratory results were collected within the University Hospitals Leuven laboratory information system. Results from tests performed on clinical samples were retrieved from the University Hospitals information system as well.
Sampling strategy	We aimed primarily to test whether it was practically feasible to use ambient air sampling of community settings to assess the types of pathogens being transmitted within the community over a winter season. The respiratory pathogens we targeted (n=29) reflect a large number of pathogens that frequently cause respiratory infections in the region, as well as some highly relevant but rare pathogens (e.g MERC, C burnetti). They are the pathogens contained in the respiratory panel with which our team had ample experience in clinical settings. We added a second qPCR for SARS-CoV)2 (TaqPath) because of the epidemiological significance of that pathogen, the fact that we had experience analysing air samples on that platform, and the fact that is was more sensitive for COVID-19 than the respiratory panel. The study period (October to April) and the number of community settings (21) and samples (341) are a reflection of the practical constraints of collecting the samples with a small study team, rather than a formal assessment of the required sample size.<br>We samples the air for two hours unless site specific schedules required shorter sampling (e.g. lunch time in schools). We measured environmental parameters such as CO2 and humidity either manually or using a remote climate sensor with internet-connection.
Data collection	Data collection occurred on paper for environmental/behavioural parameters (excluding continuously monitored environmental factors - see above). Laboratory test results were collected in the University Hospitals Leuven laboratory information system (see

above). All air samples were collected by trained study nurses/medical doctors or laboratory technicians (all of whom are part of the study team). Clinical sample collection invariably took place by qualified healthcare professionals within a tertiary care setting.

Timing and spatial scale	Sampling sites spanned 21 community settings in and around Leuven, Belgium. Sampling took place between 10-2021 and 04-2022. From January 17 onwards, three air filtration devices were present in one of three nursery locations: location 2. On the first 7 days of air filtration in this location, the air was sampled without filtration, filtered for several hours, then sampled again with active and carbon filter-based device with a clean air delivery rate of 333m3/h. From February 7 onwards, three Philips 3000i (Philips inc) devices were additionally placed in location 3. This is another HEPA and carbon-based device. From this moment onwards, sampling took place concurrently in all three locations. On Mondays, air filtration started after the completion of 2 hours of sampling. Air filtration then continued uninterrupted for 96 hours. On Wednesdays and Fridays, sampling was repeated in each location, again for 2 hours per day. Air filtration was discontinued after sampling on Fridays.<br>As previously stated, we aimed primarily to test whether it was practically feasible to use ambient air sampling of community settings to assess the types of pathogens being transmitted within the community over a winter season. The duration of the study aimed to span a winter season. The spatial scale (in and around Leuven, Belgium) and frequency (few times per week, with necessary interruptions during e.g school holidays) reflect the need for a small study team to collect all samples within an acceptable operational burden.
Data exclusions	When describing pathogen detection patterns across sampling sites, age groups, and time, we considered each of the 29 pathogens separately. Only the TaqPath qPCR was considered for SARS-CoV-2, to avoid duplication and because it is more sensitive. The TaqPath qPCR was not performed on 35/341 samples between January 3rd and 14th due to financial constraints. The TaqPath SARS-CoV-2 result was missing for one sample and the respiratory panel for two samples due to failed transport between labs. When analysing the influence of pathogen, host, behavioural and environmental/building related factors on bioaerosol load, we excluded pathogens with less than 10 positieve qPCR results after grouping them – to increase statistical power – as follows: human parainfluenza virus 1 to 4 under 'parainfluenza viruses'; Human coronaviruses 229E, HKU-1, NL63, and OC43 under 'other coronaviruses'. Supplementary table 3 lists the missing environmental/building related and behavioural factors and how the missing data was handled. Supplementary Methods describes the procedure for imputing the missing variables.<br>For filtration, all data-points from January 17th onwards were included in the main analyses assessing the influence of pathogen, host, behavioural and environmental/building related factors on bioaerosol load.<br>For the interventional sub-study, only samples from February 7th onwards were included. This was done because we wanted to (visually) compare the influence of filtration on pathogen load in the 3 subgroups (nursery locations 1-3).
Reproducibility	We ran multiple, complementary analysis techniques to verify results: pathogen detection AND pathogen concentration as outcomes, logistic regression + mixed logistic regression + generalized estimating equations for the former and linear regression + mixed linear regression for the latter. After backward elimination (for each model), we removed observations with imputed variables to confirm the results. We ran pathogen specific linear and logistic regression models with the significant covariates of the corresponding main models, to confirm consistency of the findings. We also used a different input for SARS-CoV-2 results (respi panel qPCR instead of TaqPath PCR to verify the main model outcomes). With regards to filtration, we used complementary analyses: adding filtration presence as a variable in the mail models, but also analysing the nursery setting as an interventional substudy with repeated sampling in 3 different groups.<br>All models pointed at the same factors being the main determinants of the ambient air bioaerosol load: the period of sampling, the pathogen, ventilation (in the form of its proxy - CO2 concentration - and/or as natural ventilation), and air filtration.<br>The association between air and clinical samples was limited, but was clearly present for SARS-CoV-2 and less clearly - for Influenza A and Enterovirus D68.<br>While we continue to conduct studies using similar methodologies, we have not performed exactly the same analyses on any of these extensions at this point in time.
Randomization	Randomization was not applicable to this observational study.
Blinding	Blinding was not applicable to this observational study.

Did the study involve field work? ☒ Yes ☐ No

Field work, collection and transport

Field conditions	The focus of the study was the indoor environment.<br>We logged host, behavioural, environmental/building related parameters for each sample. We also logged the height at which each sampler was placed.<br>The cofactors we took into account were the following:<br>the number of attendees, attendee density (number of attendees divided by room volume), sampling duration, mask wearing, vocalisation (voice use), natural ventilation (opening of doors and windows), portable air filtration, presence of mechanical ventilation, local COVID-19 incidence, indoor CO2 concentration, temperature and relative humidity.
Location	All sampling sites were within a 5 km radius from the town hall of Leuven, Belgium. No exact coordinated can be given on the specific community settings involved because no agreement has been provided by their management for the publication of their identities.
Access & import/export	NA
Disturbance	Only human attendants. Sampling took place after the management of each institution had agreed to take part in the study.

Reporting for specific materials, systems and methods

We require information from authors about some types of materials, experimental systems and methods used in many studies. Here, indicate whether each material, system or method listed is relevant to your study. If you are not sure if a list item applies to your research, read the appropriate section before selecting a response.

Materials & experimental systems

n/a	Involved in the study	
☒	☐	Antibodies
☒	☐	Eukaryotic cell lines
☒	☐	Palaeontology and archaeology
☒	☐	Animals and other organisms
☒	☐	Clinical data
☒	☐	Dual use research of concern

Methods

n/a	Involved in the study	
☒	☐	ChIP-seq
☒	☐	Flow cytometry
☒	☐	MRI-based neuroimaging
